# Longitudinal blood glucose level and increased silent myocardial infarction: a pooled analysis of four cohort studies

**DOI:** 10.1186/s12933-024-02212-3

**Published:** 2024-04-18

**Authors:** Mianli Xiao, Markku A. Malmi, Douglas D. Schocken, Janice C. Zgibor, Amy C. Alman

**Affiliations:** 1 College of Public Health, University of South Florida, Tampa, FL USA; 2 Department of Medicine, Duke University Medical Center, Durham, NC USA

**Keywords:** Longitudinal fasting glucose (FG), Silent myocardial infarction, Diabetes, Intra-individual FG variability

## Abstract

**Background:**

Fasting glucose (FG) demonstrates dynamic fluctuations over time and is associated with cardiovascular outcomes, yet current research is limited by small sample sizes and relies solely on baseline glycemic levels. Our research aims to investigate the longitudinal association between FG and silent myocardial infarction (SMI) and also delves into the nuanced aspect of dose response in a large pooled dataset of four cohort studies.

**Methods:**

We analyzed data from 24,732 individuals from four prospective cohort studies who were free of myocardial infarction history at baseline. We calculated average FG and intra-individual FG variability (coefficient of variation), while SMI cases were identified using 12-lead ECG exams with the Minnesota codes and medical history. FG was measured for each subject during the study’s follow-up period. We applied a Cox regression model with time-dependent variables to assess the association between FG and SMI with adjustment for age, gender, race, Study, smoking, longitudinal BMI, low-density lipoprotein level, blood pressure, and serum creatinine.

**Results:**

The average mean age of the study population was 60.5 (sd: 10.3) years with median fasting glucose of 97.3 mg/dL at baseline. During an average of 9 years of follow-up, 357 SMI events were observed (incidence rate, 1.3 per 1000 person-years). The association between FG and SMI was linear and each 25 mg/dL increment in FG was associated with a 15% increase in the risk of SMI. This association remained significant after adjusting for the use of lipid-lowering medication, antihypertensive medication, antidiabetic medication, and insulin treatment (HR 1.08, 95% CI 1.01–1.16). Higher average FG (HR per 25 mg/dL increase: 1.17, 95% CI 1.08–1.26) and variability of FG (HR per 1 sd increase: 1.23, 95% CI 1.12–1.34) over visits were also correlated with increased SMI risk.

**Conclusions:**

Higher longitudinal FG and larger intra-individual variability in FG over time were associated in a dose–response manner with a higher SMI risk. These findings support the significance of routine cardiac screening for subjects with elevated FG, with and without diabetes.

**Supplementary Information:**

The online version contains supplementary material available at 10.1186/s12933-024-02212-3.

## Highlights

1. Higher fasting glucose correlated with silent myocardial infarction (SMI): Each 25 mg/dL increase in glucose links to a 15% rise in SMI risk, independent of medication use.
2. Consistent pattern: The longitudinal study involving 24,732 individuals revealed a linear relationship between fasting glucose levels and SMI occurrence over a 9-year follow-up.
3. Variability matters: Greater intra-individual fasting glucose variation elevated SMI risk, reinforcing the need for stable glucose levels.
4. Clinical significance: These results emphasize routine cardiac screening, particularly for individuals with elevated glucose, as a crucial approach for preventing SMI and promoting heart health.

**Supplementary Information:**

The online version contains supplementary material available at 10.1186/s12933-024-02212-3.

## Background

Myocardial infarctions (MIs) that are asymptomatic or with minor and atypical symptoms are often diagnosed through routine electrocardiographic (ECG) examination. Silent myocardial infarction (SMI) is characterized by ECG evidence of MI without a prior history of MI, and makes up one-quarter to one-half of all MI cases [1, 2]. SMI increases the risk of subsequent heart failure [3], sudden cardiac death [4], and all-cause mortality [5].

Diabetes and elevated blood glucose are correlated with increased cardiovascular events. MI occurs twice as frequently in people with diabetes compared to those without diabetes [6, 7]. Having higher HbA1c and insulin use are also associated with an elevated risk of SMI incidence in people with type 2 diabetes [8]. In a cross-sectional study, individuals with impaired fasting glucose (IFG) had a higher prevalence of SMI [9]. However, in a longitudinal cohort study of elderly adults, only subjects with fasting glucose (FG) in the diabetic range but not IFG (compared to normal glucose) had increased SMI risk after adjusting for covariates like the use of antihypertensive and lipid-lowering medication and total cholesterol [10]. Uncertainty surrounds the relationship between the prediabetic state and the increased risk of SMI. Most prior studies examined the association between diabetes status and SMI, but limited evidence exists examining the association between FG in either the diabetic or prediabetic range or assessing the dose–response between FG and incidence of SMI. Moreover, FG is not a stable variable and could be influenced by factors such as age [11], BMI [12], diet [13], the use of oral anti-diabetic medication, and insulin. Given the variability of FG and this uncertain relationship, it is essential to evaluate the association of longitudinal glucose across the full range of values and SMI in a large population. The objective of our study is to investigate the association between repeatedly measured FG and the incidence of SMI in a pooled analysis from four prospective cohort studies.

## Methods

### Study population

This study combined individual data from four cohort studies: the Atherosclerosis Risk in Communities (ARIC) Study [14], the Cardiovascular Health Study (CHS) [15], the Multi-Ethnic Study of Atherosclerosis (MESA) Study [16], and the Health, Aging and Body Composition (HABC) Study [17]. Subjects were excluded from the study if they had abnormal ECG results, clinical MI, coronary artery bypass graft, or coronary angioplasty history at baseline, missing data in ECG or FG, or did not attend any follow-up visits.

ARIC is a community-based prospective cohort study that was started in 1987 to examine the risk factors and evolution of CVD. At the baseline visit (1987–1989), a total of 15,792 individuals aged between 45 and 64 from four US communities were enrolled. Follow-up visits were conducted every three years: 1990–1992 (visit 2), 1993–1995 (visit 3), 1996–1998 (visit 4), and visit 5 was conducted from 2011–2013. Data from the baseline period (1987–1989) through 2013 (visit 5), were used in the present investigation. After exclusion, data from a total number of 13,137 individuals were enrolled for analysis.

CHS is a cohort study designed to assess the risk factors of CVD in the elderly. The study recruited 5888 individuals aged 65 or older from four U.S. communities (Maryland, North Carolina, Pennsylvania, and California), and performed annual clinical examinations between 1989 and 1999, followed by semi-annual phone calls. The present study included data obtained from baseline (1989–1990 for the original cohort and 1992–1993 for the additional recruitment) through 1999, while in-person ECG examinations were conducted.

MESA is a prospective, population-based observational cohort study that included 6814 subjects from four different racial/ethnic groups (White, Black, Hispanic, and Asian) and 6 medical centers in the U.S., aged 45 to 84, and free of CVD at the time of enrollment (2000–2002). Follow-up visits were conducted every two years, and ECG examinations were conducted during baseline visits and visit 5 (2010–2011). Data obtained from the first 5 exams (2000–2011) was used for the present study.

HABC is a longitudinal cohort study initiated in 1997–1998. The study enrolled 3075 men and women aged 70–79 at baseline, with 45% of the women and 33% of the men identified as African-American. All participants were randomly recruited as a sample of Medicare beneficiaries from two clinical centers in Pittsburgh, PA, and Memphis, TN. We included data from the first four annual visits (1997–2001) and ECG examinations were conducted in baseline and visit four.

We selected these cohorts because they are all prospective cohort studies designed to investigate the incidence and risk factors of CVD with repeated FG measurement and available ECG exams to determine SMI status.

We excluded the following participants: (1) subjects with missing or incomplete ECGs; (2) subjects with missing data on FG at all visits; (3) subjects with CVD history at enrollment which were defined as the presence of ECG evidence of MI, a self‐reported history of physician‐diagnosed MI, coronary artery bypass surgery, and coronary angioplasty. After all exclusions, the final analysis cohorts consisted of 13,137 ARIC study participants, 4850 CHS participants, 4271 MESA participants, and 2259 HABC participants.

All participants provided written informed consent at enrollment, and all four studies were approved by the institutional review boards at corresponding participating institutions. The institutional review board at USF deemed this study non-human subjects research.

### ECG examination and diagnostic criteria for SMI

Each participant had a standard 12-lead resting ECG taken at the study examination, at least 1 h after consuming any coffee or smoking, using MAC PC ECG equipment (Marquette Electronics, Milwaukee, WI). Two medical professionals who had undergone training in interpreting ECGs following the Minnesota code (MC) and were blinded to the outcome status read all ECGs. A senior practitioner, not involved in the initial review, made the final assessments regarding any discrepancies between the two professionals.

SMI was defined as ECG evidence of new MI during follow‐up visits that were not present at enrollment and in the absence of recorded clinical MI (including diagnosis and/or hospitalization for an MI). The newly introduced MC ECG classifications were used to characterize ECG evidence of MI as a major Q/QS wave abnormality (MC 1.1 or 1.2) or minor Q/QS wave abnormality (MC 1.3) alongside a major STT abnormality (MC 4.1, MC 4.2, MC 5.1, or MC 5.2) [18].

### Longitudinal FG and other covariates

Our primary exposure was longitudinal measures of FG. Covariates included age, gender, race, smoking status (current, former, never), body mass index (BMI), blood pressure, low-density lipoprotein (LDL), serum creatinine, the use of lipid-lowering medication, antihypertensive medication, antidiabetic medication, and insulin treatment. All independent variables, except for age, gender, race, and smoking were measured at each follow-up visit over the length of the studies. Additional file 1: Fig. S1 illustrates the time points at which FG and ECG measurements were taken. For the majority of the FG measurements, they align with the timing of ECG exams, with one exception noted in the MESA study. In the MESA study, ECG assessments were only conducted at the baseline and Visit 5. Missing covariate data were imputed using the value from the nearest visit for the same individual.

### Statistical analysis

The primary outcome of our study was the incidence of SMI which was interval censored between two sequential visits. The exposure and covariates were time-dependent variables, therefore, we used Cox proportional-hazards models with time-dependent variables to estimate the association between time-varying FG and SMI, with adjustment for potential confounders, including age, gender, race, study, smoking status, diastolic blood pressure (DBP), LDL, blood creatinine, and BMI (Model 1). In Model 2, we further adjusted for the use of lipid-lowering medication, antihypertensive medication, antidiabetic medication, and insulin treatment as covariates to see if adjustment for treatments had any further impact on the association between FG and SMI. We applied the penalized splines for FG in the Cox regression model to test for a potential non-linear association between FG and the incidence of SMI. Subgroup analyses were conducted by gender and by age stratified at 65 years at baseline. We also performed sensitivity analyses by examining the association taking the average FG over time and the intra-individual variability of FG over time of each individual as the exposure. The variability of FG was calculated as the coefficient of variation (CV, sd/mean across visits). All the analysis work was conducted in R with *Survival* package version 3.4–0, and *rms* package version 6.3–0.

## Results

After exclusion criteria were applied, there were 13,137 individuals from the ARIC study, 4850 individuals from the CHS study, 4486 individuals from the MESA study, and 2259 individuals from the HABC study available for analysis. Among the included subjects, the median follow-up time was 9.1 years for ARIC, 8.8 years for CHS, 9.4 years for MESA, 3.0 years for HABC, and 9.0 years across all cohorts. The baseline characteristics of the study population are shown in Table 1. As observed from 24,732 subjects, the median FG was 97.3 (25th–75th: 90–107) mg/dL at baseline. During the follow-up period, 357 SMI incidence cases (incidence rate, 1.29 events per 1000 person-years) and 1036 MI incident cases (incidence rate, 3.74 events per 1000 person-years) were observed.

**Table 1:** Baseline characteristics of the study population

Measurements, Mean (SD)/N (%)	Overall	ARIC	CHS	MESA	HABC
Population	(N = 24,732)	(N = 13,137)	(N = 4850)	(N = 4486)	(N = 2259)
Event counts	357 (1.5%)	139 (1.1%)	77 (1.5%)	104 (2.2%)	37 (1.7%)
Follow-up time, Median [IQR]	8.97 [6.85, 10.28]	9.11 [8.77, 23.34]	8.80 [5.92, 8.95]	9.40 [9.14, 9.73]	3.01 [2.95, 3.10]
Age at baseline, yr	60.5 (10.3)	54.0 (5.72)	72.1 (5.51)	60.3 (9.57)	73.5 (2.84)
Gender					
Female	14,067 (56.9%)	7433 (56.6%)	2950 (60.8%)	2398 (53.5%)	1286 (56.9%)
Male	10,665 (43.1%)	5704 (43.4%)	1900 (39.2%)	2088 (46.5%)	973 (43.1%)
Race					
Black	6134 (24.8%)	3256 (24.8%)	768 (15.8%)	1192 (26.6%)	918 (40.6%)
White	17,075 (69.0%)	9881 (75.2%)	4050 (83.5%)	1803 (40.2%)	1341 (59.4%)
Other	1523 (6.2%)	0 (0%)	32 (0.7%)	1491 (33.2%)	0 (0%)
Smoking status					
Current	4635 (18.7%)	3281 (25.0%)	589 (12.1%)	554 (12.3%)	211 (9.3%)
Former	8751 (35.4%)	4183 (31.8%)	1937 (39.9%)	1646 (36.7%)	985 (43.6%)
Never	11,346 (45.9%)	5673 (43.2%)	2324 (47.9%)	2286 (51.0%)	1063 (47.1%)
BMI	27.5 (5.09)	27.6 (5.30)	26.6 (4.10)	28.3 (5.37)	27.4 (4.80)
Fasting glucose (mg/dL), Median [IQR]	97.3 [90.0, 106.9]	99.0 [93.0, 107.0]	101.0 [94.0, 111.0]	89.0 [82.0, 98.0]	94.0 [87.0, 104.0]
Systolic blood pressure (mmHg)	126 (20.8)	121 (18.3)	140 (20.6)	125 (20.4)	135 (20.5)
Diastolic blood pressure (mmHg)	72.6 (11.1)	73.6 (11.0)	71.0 (11.5)	71.9 (10.1)	71.3 (11.6)
LDL (mg/dL)	131 (37.4)	137 (39.1)	130 (35.4)	117 (31.3)	123 (33.7)
HDL (mg/dL)	52.8 (16.5)	52.1 (17.0)	55.2 (15.7)	51.2 (14.9)	55.1 (16.8)
Medication use					
Antihypertensive medication	8178 (33.1%)	3415 (26.0%)	2106 (43.4%)	1523 (34.0%)	1134 (50.2%)
Lipid-lowering drugs	1332 (5.4%)	148 (1.1%)	221 (4.6%)	716 (16.0%)	247 (10.9%)
Oral Antidiabetics	1118 (4.5%)	371 (2.8%)	259 (5.3%)	329 (7.3%)	159 (7.0%)
Insulin	480 (1.9%)	233 (1.8%)	97 (2.0%)	64 (1.4%)	86 (3.8%)

LDL: low-density lipoprotein, HDL: high-density lipoprotein

Data from four studies—the Atherosclerosis Risk in Communities (ARIC) Study, the Cardiovascular Health Study (CHS), the Multi-Ethnic Study of Atherosclerosis (MESA) study, and the Health, Aging and Body Composition (HABC) Study are shown

**Fig. 1:**
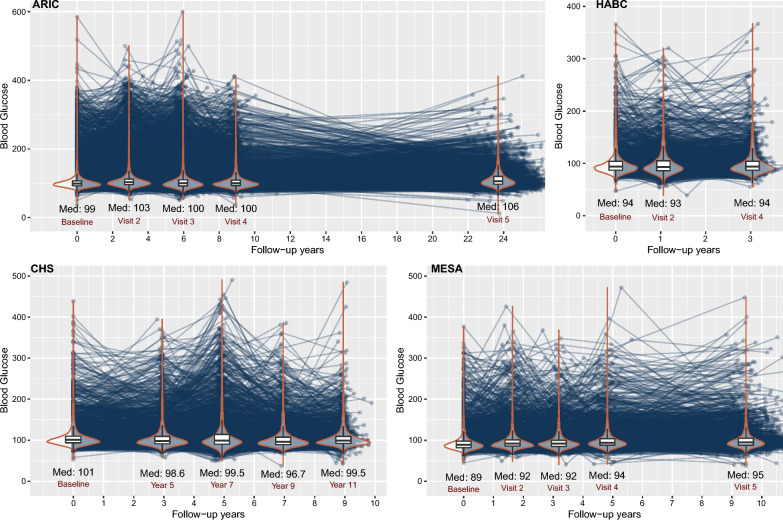
The longitudinal blood glucose level over time in four studies. Med: median

Figure 1 shows the change in FG of each individual over visits. The median FG slightly increased from baseline to visit 5 in ARIC (99–106 mg/dL) and MESA (89–95 mg/dL) but remained stable in the HABC and CHS studies. Subjects without diabetes at baseline tend to maintain stable FG over time (mean CV = 0.06), but subjects with high FG, especially those over 150 mg/dL, experienced significant fluctuations over time (mean CV = 0.25).

**Fig. 2:**
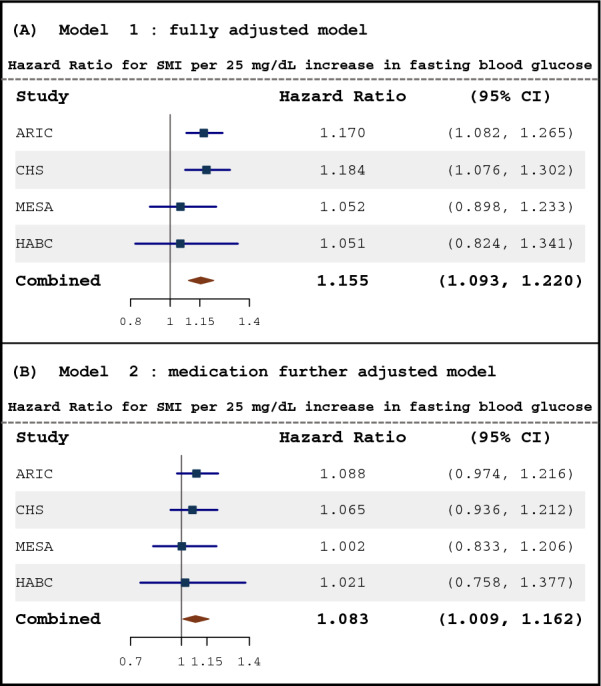
The correlation between blood glucose and incident silent myocardial infarction. The hazard ratios for SMI with the corresponding 95% confidence intervals as a spline function of blood glucose from Cox proportional hazard regression models adjusted for covariates in 2 models. Model 1 adjusted for age, gender, race, studies, smoking status, diastolic blood pressure, blood low-density lipid, BMI, and blood creatinine; Model 2 additionally adjusted for the use of lipid-lowering medication, antihypertensive medication, antidiabetic medication, and insulin treatment

The association between longitudinal FG and SMI in the four cohort studies and pooled population in both Model 1 and Model 2 is shown in Fig. 2. Hazard ratios (HRs) and 95% confidence intervals (CIs) of FG (per 25 mg/dL increase) were estimated by adjusting for potential confounders including age, gender, race, study, smoking status, and time-varying DBP, BMI, LDL, and blood creatinine. Repeated measures of FG were positively correlated with an increased risk for incident SMI in the four cohort studies but the HRs were only statistically significant in ARIC (HR: 1.17, 95% CI 1.08–1.27) and CHS (HR: 1.18, 95% CI 1.08–1.30) studies but not in MESA (HR: 1.05, 95% CI 0.90–1.23) and HABC (HR: 1.05, 95% CI 0.82–1.34) studies. The HR for SMI in the combined population from the four studies was 1.16 (95% CI 1.09–1.22), which indicated for each 25 mg/dL increment in FG there was a 15% increase in the risk of SMI. In model 2, after adjusting for the use of multiple medications including anti-diabetes, insulin, antihypertensive, and lipid-lowering medications, the association between FG and incident SMI was attenuated. Each 25 mg/dL increment in FG was associated with an 8% (95% CI 9–16%) increase in risk of SMI in the combined population after further adjustment for medication use.

**Fig. 3:**
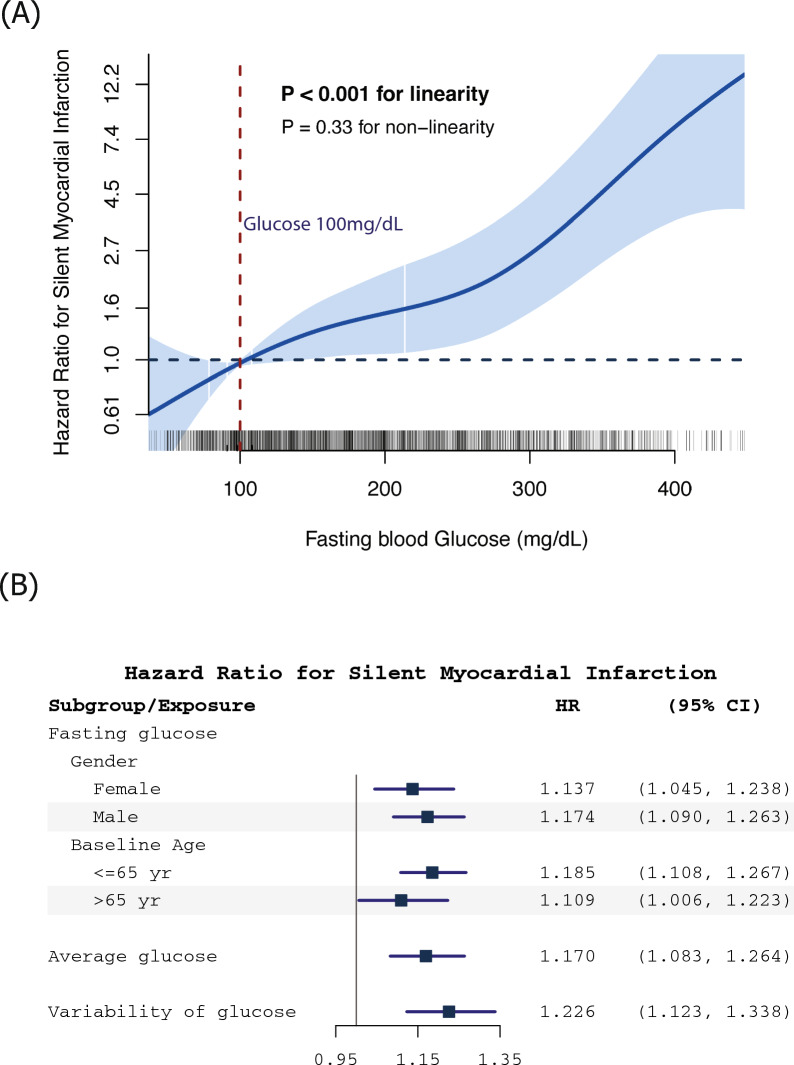
**A** The dose–response of fasting blood glucose on the incidence of silent myocardial infarction. This association was estimated after adjusting for age, gender, race, studies, smoking status, diastolic blood pressure, blood low-density lipoprotein, BMI, and blood creatinine. **B** Subgroup analysis and sensitivity analysis between glucose and the incidence of silent myocardial infarction. All hazard ratios and 95% confidence intervals are estimated in fully adjusted models (adjusted for age, gender, race, studies, smoking status, diastolic blood pressure, blood low-density lipid, BMI, and blood creatinine). ¶: repeated measures fasting glucose (per 25 mg/dL); †: average glucose over time of each subject (per 25 mg/dL); ‡ variability of glucose (per 1 sd)

We performed a Cox regression model with a penalized spline to test for a non-linear association and dose–response between FG and SMI (Fig. 3A). The association was linear (*P* < 0.001) and the non-linear term was not statistically significant (*P* = 0.33).

The results of subgroup analysis according to gender and age at baseline are shown in Fig. 3B. The positive correlation between FG and SMI risk remained significant regardless of gender or age. For sensitivity analyses of the average FG over follow-up, in the combined population, each additional 25 mg/dL increase in average FG was associated with a 17% (95% CI 8–26%) increased risk of SMI. An elevated variability in the longitudinally measured FG of each subject was also correlated with increased SMI risk, and each 1 sd increment in glucose variability was associated with a 23% (95% CI 12–34%) increased risk of SMI. HRs of subgroups and sensitivity analyses were adjusted for age, gender (except for analyses stratified by gender), race, study, smoking status, and time-varying DBP, BMI, LDL, and serum creatinine.

## Discussion

We observed an upward trend in FG only within the ARIC and MESA studies. The disparity in baseline age may account for the observed difference in FG change. In the ARIC and MESA studies, the mean baseline age was 54.0 (sd 5.72) and 60.3 (sd 9.57) years, respectively, while participants in the CHS and HABC studies had an average baseline age of 72.1 (sd 5.51) and 73.5 (sd 2.84) years. Existing literature has shown that fasting plasma glucose levels exhibit a continual increase from the age of 30, plateauing around 65 or 70 years old [11, 19, 20].

In this study which pooled data from four prospective cohort studies in the United States, involving 24,732 middle-aged and elderly subjects with a median follow-up of 9 years, we found that higher FG, measured longitudinally, was significantly associated with a higher risk of SMI in a dose–response manner throughout the whole range of FG. The association between FG and SMI is not independent of the use of anti-diabetes medication and insulin, as the adjustment for the use of anti-diabetes medication and insulin attenuated the association. However, the association was not undermined by adjusting for the use of antihypertensive or lipid-lowering medication and remained statistically significant after considering the use of four types of medication. The average levels and variability of FG over time were also positively associated with an increased risk of SMI.

The increased risk of major CVD among diabetes patients including type 1 (T1D) and type 2 diabetes (T2D) is widely known [6, 21]. The pathophysiology that links diabetes and cardiovascular complaints is endothelial cell dysfunction induced by hyperglycemia, excess free fatty acid release, and insulin resistance [22]. A hypothesis posits that hyperglycemia is a key factor in the development of diabetes complications [23]. This mechanism is thought to occur through the increased production of a specific type of reactive oxygen species called superoxide in the mitochondria, which activates certain pathways (such as the polyol, hexosamine, protein kinase C, and advanced glycation end-product pathways). These pathways contribute to the development of microvascular and macrovascular complications [24]. Microvascular and macrovascular diseases are connected, as microvascular diseases could promote the development of atherosclerosis in macrovascular vessels such as coronary arteries through mechanisms like hypoxia and alterations in the vasa vasorum [25, 26].

Several studies investigated the association between glycemic control and cardiovascular complications among diabetes patients, with most supporting the beneficial effect of glycemic control on CVD. Stratton et al. prospectively observed 3642 T2D patients and found that each 1% reduction in updated mean HbA1c was associated with a 14% reduction in the risk of MI (8% to 21%, *P* < 0.001) [27]. Results from a randomized controlled trial showed that after an average of 6.5 years of treatment and an additional average of 17 years of follow-up, intensive therapy (near normoglycemia) significantly reduced the risk of any CVD event by 42% (95% CI 9–63%) compared to conventional therapy [28]. A study conducted by Matuleviciene-Anängen et al. [29] found that improved glycemic control in a large group of individuals with T1D (N = 33,170) was associated with a reduction in acute MI, CVD death, and overall CVD risk.

Diabetes is characterized not only by hyperglycemia, but also by significant fluctuations in glycemic levels, and a recent study indicated fluctuations in glucose levels are linked to increased levels of oxidative stress, impaired function of the blood vessel lining, and inflammation, which are all known to contribute to the development of macrovascular complications [29]. Our study revealed that larger intra-individual variability in FG was also associated with an elevated risk of SMI. These findings are consistent with results from a cohort study that enrolled 3769 young adults and follow-up for up to 30 years, they reported higher intra-individual FG variability during young adulthood was associated with increased risk of CVD and mortality [30].

In our study, we found a linear relationship between FG and SMI in all participants, not just in those with defined diabetes. A meta-analysis summarized data from 95,783 subjects and reported that compared with a 2-h glucose level of 75 mg/dL, having a fasting and 2-h glucose level of 110 mg/dL (prediabetes) was associated with a 33% increased risk of cardiovascular events [31]. Other studies investigated the association between FG and SMI, but those studies drew no clear conclusion, particularly for values below the diabetes diagnosis threshold. Results from a cross-sectional study using data from MESA noted that compared with individuals with normal FG, those with impaired FG had a higher prevalence of SMI (Odds Ratio [OR]: 1.60, 95% CI 1.0–2.5) after adjusting for multiple confounders [9]. The Fenofibrate Intervention and Event Lowering in Diabetes (FIELD) study reported increased HbA1c to be a predictor for SMI but the result was not statistically significant (OR: 1.04 95% CI 0.94–1.16) [8].

Prediabetes is an unstable condition that can progress into T2D or return to normal glucose regulation. Identification of SMI remains challenging given the asymptomatic or atypical nature and can only be captured by routine ECG or other cardiac imaging examinations. The instability of prediabetes and the challenge in SMI identification may account for the difficulty in determining the relationship between them. However, previous research indicated that in healthy subjects, impaired FG combined with SMI was associated with a poor prognosis [32]. Given that multiple studies found an increase in subsequent cardiovascular risk following SMI [5, 33, 34], their significance should not be underestimated.

This study had certain limitations, one of which is the use of 12-lead ECG to diagnose SMI, which has lower accuracy in detecting SMI than methods like cardiac magnetic resonance (CMR) [34]. Yet the common use of ECG and standardized protocols for interpretation make it possible to pool data from multiple studies and provide stronger power to detect an association. Although ECGs may not be as accurate as CMR in identifying SMI, they are a cost-effective way to screen for potential cardiovascular endpoints [35]. Additionally, using FG alone is not a superior method for evaluating long-term blood sugar control compared to HbA1c. However, taking multiple measurements of FG and averaging them over time can help address this issue.

## Conclusions

Increased longitudinal FG, average FG, and intra-individual variability of FG over time were all associated with an elevated risk of SMI during follow-up in this study population after adjusting for multiple confounders including medication use. Given the asymptomatic or atypical nature of SMI, our findings support the significance of routine cardiac screening, especially for subjects with diabetes or impaired FG since the risk of having SMI is elevated with the increase in FG.

### Supplementary Information

**Additional file 1. **Time points of measurement of fasting glucose and ECG in the ARIC, MESA, Health ABC, and CHS cohorts.

## Data Availability

The datasets generated and/or analyzed during the current study are available in the Biologic Specimen and Data Repository Information Coordinating Center (BIOLINCC).
