# Supplementary figures and images for: Longitudinal blood glucose level and increased silent myocardial infarction: a pooled analysis of four cohort studies
Source: Cardiovasc Diabetol. 2024 Apr 18;23:130. doi: 10.1186/s12933-024-02212-3 (PMC11027351; doi:10.1186/s12933-024-02212-3)

ARIC

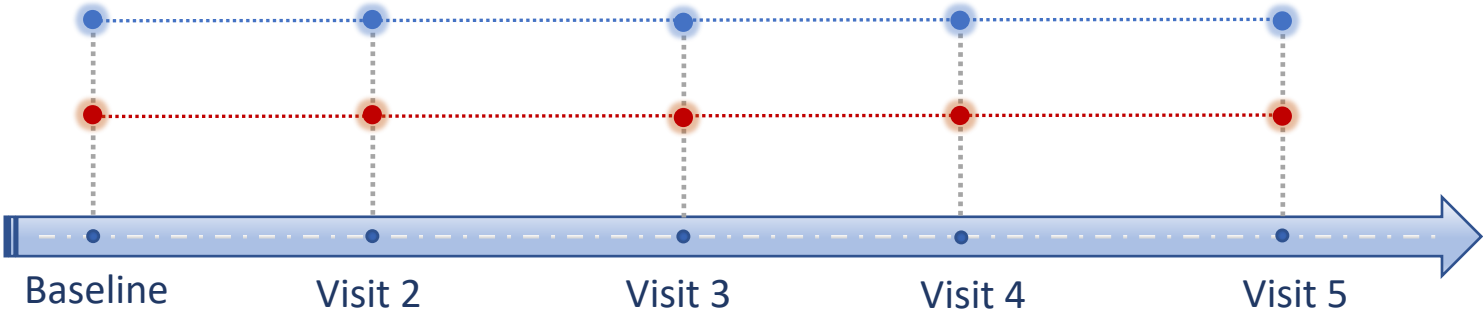

MESA

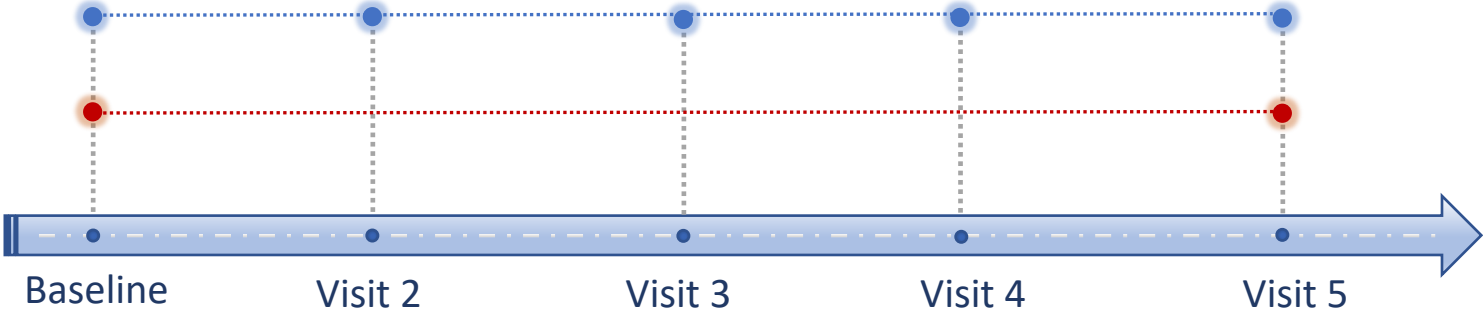

Health ABC

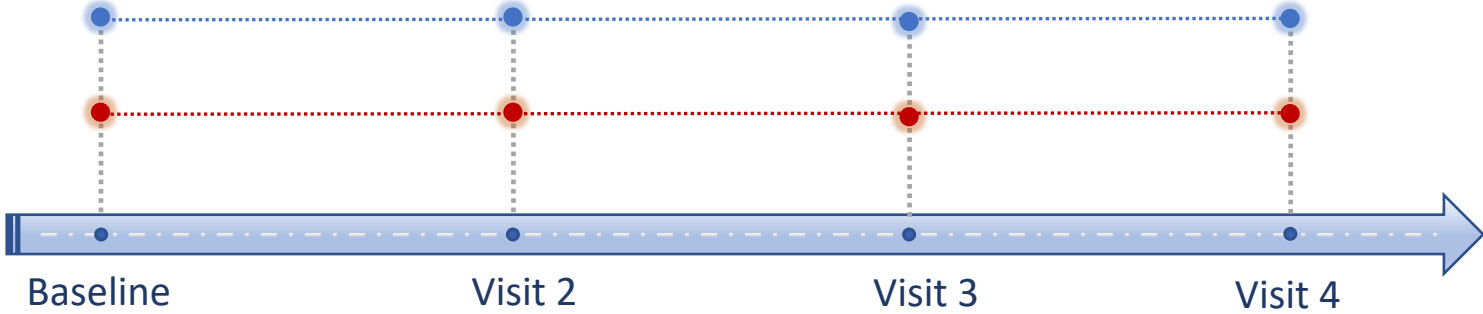

CHS

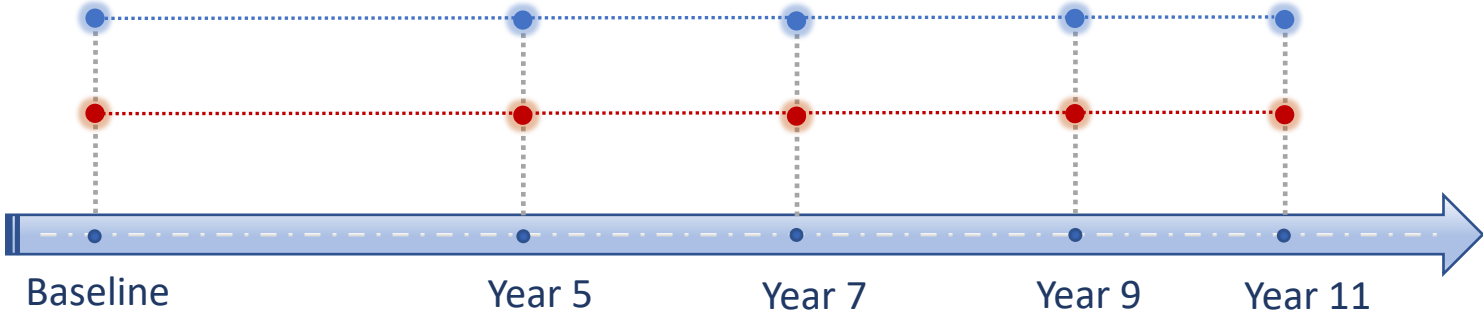

● Fasting glucose ● ECG

Supplement: Supplementary file 1 — Additional file 1. Time points of measurement of fasting glucose and ECG in the ARIC, MESA, Health ABC, and CHS cohorts. [file 12933_2024_2212_MOESM1_ESM.pdf]
